# Bmi-1 expression predicts prognosis in squamous cell carcinoma of the tongue

**DOI:** 10.1038/sj.bjc.6605544

**Published:** 2010-02-09

**Authors:** V Häyry, L K Mäkinen, T Atula, H Sariola, A Mäkitie, I Leivo, H Keski-Säntti, J Lundin, C Haglund, J Hagström

**Affiliations:** 1 Department of Otolaryngology and Head and Neck Surgery, Helsinki University Central Hospital, PO Box 220, Haartmaninkatu 4E, Helsinki 00290 HUS, Finland; 2 Department of Developmental Biology, Institute of Biomedicine, University of Helsinki, PO Box 63, Haartmaninkatu 8, Helsinki 00014, Finland; 3 Department of Pathology, Haartman Institute, University of Helsinki, PO Box 21, Haartmaninkatu 3, Helsinki 00014, Finland; 4 Department of Oncology, Helsinki University Central Hospital, PO Box 180, Haartmaninkatu 4, Helsinki 00290 HUS, Finland; 5 Department of General Surgery, Helsinki University Central Hospital, PO Box 340, Haartmaninkatu 4, Helsinki 00290 HUS, Finland; 6 Department of Oral Pathology, Institute of Dentistry, University of Helsinki, PO Box 41, Mannerheimintie 172, Helsinki 00014, Finland

**Keywords:** Bmi-1, c-myc, Snail, oral cancer, prognosis, immunohistochemistry

## Abstract

**Background:**

The prognosis of squamous cell carcinoma of the oral tongue is poor and it would be beneficial to find prognostic markers to better adjust treatment. Bmi-1 controls cell cycle and self-renewal of tissue stem cells, transcription factor c-myc affects cell proliferation and apoptosis, and Snail regulates epithelial–mesenchymal transition. The expression of these markers has been connected to prognosis in many cancer types.

**Methods:**

Bmi-1, c-myc, and Snail expressions were studied in our material consisting of 73 primarily T1N0M0 oral tongue carcinoma patients. We compared the immunoexpressions of Bmi1, c-myc, and Snail with clinical parameters including the degree of histological differentiation, tumour size, TNM classification, depth of invasion, and resection margins. In addition, survival analyses were performed, comparing disease-free survival time with the registered protein expression of the markers mentioned above.

**Results:**

A significant correlation between Bmi-1 protein expression and recurrence (log-rank test, *P*=0.005) was detected. Snail and c-myc expression did not correlate with prognosis. Snail expression correlated with histopathological grade (Fisher's exact test, *P*=0.007) and with the invasion depth of tumours (*χ*^2^-test, *P*=0.037).

**Conclusion:**

Negative Bmi-1 immunoexpression might serve as a marker of poor prognosis in oral tongue carcinoma patients.

Squamous cell carcinoma (SCC) is the most common malignancy of the upper aerodigestive tract (Barnes *et al*, 2005). Dissemination of SCC through lymphatic routes may occur in the early course of the disease. Presence of lymph node metastasis is considered the most important tumour-related adverse prognostic factor in head and neck SCC (Ferlito *et al*, 2002a, 2002b; Howell and Grandis, 2005). In individual patients, the TNM classification does not reliably predict clinical outcome; even small T1 oral SCCs (OSCC) may have occult nodal metastasis and may potentially behave aggressively (Keski-Säntti *et al*, 2007). Identifying these patients is important for optimal treatment planning. In many studies, the histological grade of OSCC is found to be non-predictive, although some studies claim the opposite (Al-Rajhi *et al*, 2000; Keski-Säntti *et al*, 2007; Silveira *et al*, 2007; Arduino *et al*, 2008). It seems that the most important histopathological predictor of nodal metastasis and poor survival is the depth of tumour infiltration (Woolgar, 2006). It has been suggested, however, that the value of depth of infiltration in clinical decision making is limited because of poor specificity in identifying high-risk patients (Keski-Säntti *et al*, 2007).

Bmi-1 is an essential constituent of the polycomb repressive complex 1, a key epigenetic regulator. Through chromatin and histone modifications (for example, methylation), it controls the cell cycle and self-renewal of tissue stem cells (Spivakov and Fisher, 2007). Bmi-1 can influence the central tumour suppressors Rb and p53 by suppressing the p16^Ink4a^/p19^Arf^ locus (Molofsky *et al*, 2005). Bmi-1 is overexpressed in OSCC cells when compared with normal mucosa and is thought to influence cell proliferation and survival in oral carcinogenesis (Kang *et al*, 2007). Overexpression of Bmi-1 is found to correlate with poor prognosis in the non-keratinising type of nasopharyngeal carcinoma, breast, and hepatocellular carcinoma, as well as in nervous system tumours such as oligodendrogliomas and medulloblastomas, and correlation with tumour stage has been reported in non-small-cell lung cancer (Kim *et al*, 2004; Leung *et al*, 2004; Song *et al*, 2006; Häyry *et al*, 2008; Vrzalikova *et al*, 2008; Wang *et al*, 2008).

The c-myc proto-oncogene was first detected in Burkitt's lymphoma, but has later been connected to many other cancers, including breast and colon carcinomas, neuroblastomas, osteosarcomas, and melanomas (Pelengaris *et al*, 2002). C-myc is a transcription factor that activates many other genes and effects, for example, cell proliferation and apoptosis. Both Bmi-1 and c-myc are capable of immortalising certain cells *in vitro* and these factors converge on the same pathways as c-myc signalling can influence Bmi-1 activity and vice versa (Jacobs *et al*, 1999; Dimri *et al*, 2002; Gil *et al*, 2005; Guney *et al*, 2006). C-myc is also assumed to participate in oral carcinogenesis, and downregulation of c-myc mRNA has even been associated with poor prognosis (Vora *et al*, 2007; Koehn *et al*, 2008).

Snail is a zinc-finger transcription factor essential for epithelial–mesenchymal transition because it downregulates the expression of cell adhesion and basement membrane proteins, most importantly cadherins (Batlle *et al*, 2000). Thus, cells gain a fibroblast-like phenotype capable of migration and also enhanced invasiveness, as has been shown in SCC cells (Takkunen *et al*, 2006, 2008). In OSCC, the loss of cell adhesion molecules (for example, E-cadherin and *β*-catenin), the downregulation of which is linked to Snail expression, has been associated with tumour differentiation (Zidar *et al*, 2008). In OSCC, Snail immunopositivity has been detected predominantly only in the invasive front, and the overall expression has been very low (Zidar *et al*, 2008). However, no association with metastases has been observed (Mahomed *et al*, 2007), even though in another epithelial carcinoma, oesophageal SCC, Snail expression in the invasive tumour front seems to correlate with lymph node metastases, as well as with clinicopathological tumour stage (Usami *et al*, 2008).

With regard to the management of OSCC, it would be remarkably advantageous to find prognostic markers that better predict clinical outcome. For this study, we selected early-stage oral tongue SCCs (T1N0M0 and T2N0M0) that were all treated with curative intent in a fairly uniform manner (Keski-Säntti *et al*, 2006). Immunoexpression of Snail, Bmi-1, c-myc, and Ki-67 in oral tongue carcinoma tissue array blocks was studied and compared with clinical outcome.

## Materials and methods

### Patients

Patient characteristics were the same as described by Keski-Säntti *et al* (2006, 2007). In summary, clinicopathological data of 141 patients diagnosed with SCC of the oral tongue at the Helsinki University Central Hospital between 1992 and 2002 were reviewed. Only patients with tumours clinically defined as T1/T2N0, original histopathological material available for review, and clinical follow-up data of a minimum of 24 months or until death were included in the study. The dates and causes of death were provided by Statistics Finland, the national agency of population statistics. A total of 73 patients were eligible for inclusion (36 men and 37 women, median age 59 years, range 23–95 years). Of the tumours, 35 (48%) had been clinically classified as T1 and 38 (52%) as T2. All patients had undergone resection of the primary tumour. In 31 patients, there had been no further treatment primarily. A total of 42 patients underwent elective neck treatment (neck dissection: *n*=9; neck dissection+radiotherapy: *n*=32; radiotherapy: *n*=1). All patients were treated with curative intent.

The original histological sections of each patient were re-assessed and tumour grade and depth of invasion were determined by a single experienced head and neck pathologist (IL).

### Tissue array blocks

For tissue microarray blocks, we selected three different areas in two sets from normal haematoxyllin–eosin blocks from each patient to be detached with a 1 mm punch and placed into a paraffin block with a tissue manual microarrayer (BeecherInstruments Inc, Silver Spring, MD, USA). The first area was selected close to the surface epithelium, the next in the middle of the tumour, and the last at the invading front. Two parallel tissue array blocks were produced for immunostainings. In eight patients, all array spots were missing or included no tumour tissue. In these non-representative cases, we accomplished the study by staining sections from whole tumour blocks in five cases, but representative tissue was lacking for three cases.

### Immunohistochemistry

Tissue microarray-block slides and tissue slides were cut into 4 *μ*m-thick sections, deparaffinised in xylene, rehydrated through graded alcohol series, and treated in a PT-module (LabVision UK Ltd, Runcorn, UK) in Tris-HCl buffer (pH 8.5) for 20 min at 98°C and with 0.3% Dako REAL peroxidase-blocking solution (DakoCytomation, Glostrup, Denmark) for 5 min to block endogenous peroxidase. Immunostaining was performed by adding mouse monoclonal c-myc (9E10) antibody (Santa Cruz, Santa Cruz, CA, USA, diluted 1 : 400 in Dako REAL antibody diluent, Biohit, Helsinki, Finland); or mouse monoclonal Bmi-1 (ab 14389) (Abcam, Cambridge, UK, 1 : 400); or rabbit polyclonal Snail (ab17731) (Abcam, 1 : 2000); or monoclonal mouse Ki-67 Antigen (DakoCytomation, 1 : 100) for 1 h, followed by a 30 min incubation with the Dako REAL EnVision/HRP detection system. Rabbit/mouse (ENV) reagent slides were finally visualised using Dako REAL DAB+ chromogen for 10 min. Between each step, slides were washed with PBS–0.04% Tween20. Slides were counterstained with Meyer's haematoxylin and mounted in mounting medium (Aquamount, BDH, Poole, UK). In every immunohistochemical staining batch, we used a specific positive control for every antibody used. As a negative control, specimens diluted without a primary antibody were used. As negative/positive controls, we used reactive lymph node tissue, showing a negative and a positive Bmi staining pattern (Raaphorst *et al* 2000; Dutton *et al* 2007) (not shown). As a positive control for c-myc and Snail immunohistochemistry, breast and ovarian carcinoma tissue samples were used (not shown).

### Evaluation of immunostainings

Immunostainings were evaluated by two independent pathologists (JH and HS) without knowledge of clinical data. The percentage of positive tumour cells was evaluated. No positivity was graded as 0, up to 30% positive cells were graded as 1 (very low), 30–50% as 2 (low), 50–80% as 3 (moderate), and over 80% as 4 (high). Evaluation was carried out according to Buduneli *et al* (2007). Bmi-1 and Snail positivity was nuclear and c-myc scoring was carried out as both nuclear and cytoplasmic. Each patient was expected to have six spots, and for each patient we selected the highest immunoscore for further analysis.

### Statistical analysis

For categorical, non-ordered variables, cross tabulations were analysed using the *χ*^2^-test, or when the *χ*^2^-test was not valid, Fisher's exact test was used. All *P*-values are two-sided and exact. In survival analysis, an event was defined as evidence of disease in any form, including local recurrence, neck metastasis, distant metastasis, or death of the primary disease verified in autopsy.

Follow-up time was calculated from the date of first treatment (surgery) until event, and patients alive and disease free or deceased from non-tumour-related causes were censored on the last date of follow-up. Kaplan–Meier curves were plotted from follow-up data and the log-rank test was used to compare outcome between patient categories. The Cox multivariate regression analysis was used to control for confounding factors. SPSS version 15.0 software (SPSS, Chicago, IL, USA) was used.

## Results

### Bmi-1, c-myc, and Snail protein expression

**Figure 1:**
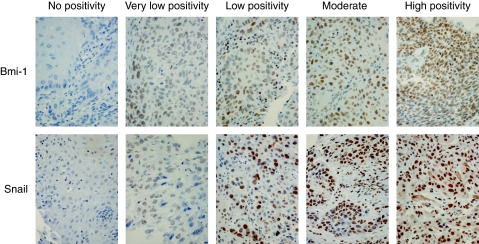
Scoring of immunohistochemical staining with Bmi-1 and Snail antibody: no cytoplasmic positivity, very low positivity is <30%, low positivity is 30–50%, moderate positivity is 51–80%, and high positivity is >80%.

**Figure 2:**
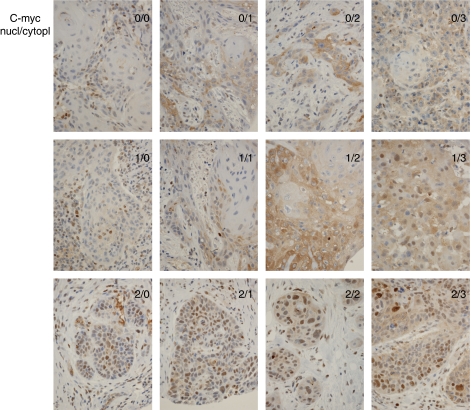
Scoring of c-myc immunopositivity in tongue carcinoma patients. Scoring was both nuclear and cytoplasmic (nucl/cytopl).

Bmi-1 protein expression was detected in 82%, nuclear c-myc expression in 63%, cytoplasmic c-myc expression in 73%, and Snail expression in 100% of all tumours. Bmi-1 expression, when present, ranged from very low to high frequency of immunoreactive cells. Nuclear c-myc expression was usually observed at a low frequency, in less than 50% of tumour cells. In only two cases was high frequency of nuclear c-myc expression found (>50%). Snail expression was observed in all samples, with the majority of tumour cells being immunoreactive. However, regarding Snail expression, two groups of patients could be distinguished, one with less than 80% immunoreactive cells and another in which virtually all tumour cells expressed Snail protein (>80%). The different levels of protein expression are summarised in Table 1 and examples of different patterns of protein expression are shown in Figures 1 and 2.

### Bmi-1 protein expression is a prognostic marker

**Figure 3:**
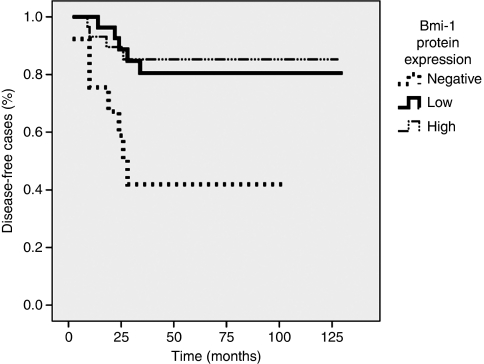
Kaplan–Meier graph of Bmi-1 expression and disease-free survival.

In a Kaplan–Meier survival analysis, a correlation between Bmi-1 protein expression and recurrence (log-rank test, *P*=0.005) was detected (Figure 3). The absence of Bmi-1 protein in the tumour cells was associated with a higher risk of recurrence. During the follow-up time of this study, only three patients died of OSCC. It is noteworthy that all three deaths occurred in Bmi-1-negative cases. The majority of patients remained disease free during follow-up. Therefore, the primary end point for survival analyses was defined as recurrence of disease. For statistical analysis, cases were grouped into three categories: Bmi-1 negative, low, and a high proportion of positive immunoreactive tumour cells. The mean disease-free time for Bmi-1-negative cases was 53 months (95% CI 29–77 months), whereas patients with tumours with a high frequency of Bmi-1-expressing cells had a mean disease-free time of 112 months (95% CI 97–126 months).

To rule out confounding factors, a multivariate analysis was also performed. In this, the depth of invasion, tumour size, margin of surgical resection, and T classification were included, along with the Bmi-1 protein expression score. In the Cox regression model, Bmi-1 expression remained the only independent covariate (*P*=0.012). The other factors were not statistically significant. The hazard ratio between absent Bmi-1 expression and high Bmi-1 expression was 5.2 (95% CI 1.5–18.3).

Although treatment of the neck does not directly reflect patient or tumour characteristics, we also analysed the prognostic impact of Bmi-1 expression with regard to the performed treatment. When patients who received an elective neck treatment and those without any elective neck treatment (observation only) were analysed independently, Bmi-1 expression was prognostic only in the group of patients in whom neck treatment was performed (log-rank test, *P*=0.004). However, the group of patients who did not receive neck treatment is smaller (28 cases analysed) than the group of patients who received elective neck treatment (42 cases); therefore, this lack of statistical significance is probably due to the small sample size. This is also the case when analysing disease-free survival times separately for recurrences in lymph nodes. Only 16 cases presented lymph node recurrence, and therefore a reliable statistical analysis is not possible.

### Prognostic significance of c-myc, Snail, and ki-67 protein expression

Survival analyses for the expression of c-myc, Snail, and Ki-67 were performed in an identical manner as that for Bmi-1, with tumour recurrence as the primary end point. For Snail, tumours were first grouped into two categories: high (>80%) and moderate (<80%) frequency of immunoreactive tumour cells. No statistically significant difference in outcome was observed between patients with high and low Snail expression (log-rank test, *P*=ns) (not shown). Similarly, tumours were grouped according to nuclear c-myc expression into negative (no expression) and positive categories.

No statistically significant difference in outcome was found between the c-myc negative and positive group (log-rank test, *P*=ns). There was no correlation between cytoplasmic c-myc expression or Ki-67 staining and survival analyses, nor in outcome (not shown).

### Clinical and histopathological correlates

The protein expression levels of all the markers (Table 1) were examined for correlations with clinical and histopathological parameters. These included degree of histological differentiation, tumour size, TNM classification, depth of invasion, and margin of resection. Snail expression was significantly lower in well-differentiated tumours, whereas in poorly differentiated tumours, Snail expression was high (Fisher's exact test, *P*=0.007). Snail protein expression was also found to correlate with depth of invasion. In cases with the highest Snail score (>80% positive tumour cells), the depth of invasion was greater (*χ*^2^-test, *P*=0.037). Finally, the prognostic significance of the above-mentioned clinicopathological features was also evaluated. In brief, no other statistically significant correlations were found.

## Discussion

In this study, we examined the value of polycomb protein and oncogene Bmi-1, as well as proto-oncogene c-myc, transcription factor Snail, and proliferation marker Ki-67 expression, in a series of 73 oral tongue carcinoma patients in relation to clinical outcome.

We found a statistically significant correlation between lack of Bmi-1 immunoexpression and poor prognosis of OSCC patients. Interestingly, Bmi-1 overexpression has been earlier reported to be constantly present in a small series of patients (*N*=10) with oral dysplastic and carcinoma tissue (Kang *et al*, 2007), but our research data consisted of 73 patients. Although high Bmi-1 overexpression is connected to poor prognosis in nasopharyngeal carcinoma (Song *et al*, 2006) and to non-small-cell lung cancer, breast carcinoma, and hepatocellular carcinoma (Vrzalikova *et al*, 2008), in our data, Bmi-1-negative tumours showed a correlation with poor prognosis. Several plausible explanations can be found for this. First, the roles of polycomb proteins are highly varied and depend on the composition of the polycomb repressive complex that Bmi-1 is a part of. The target genes and thus cellular functions such as migration, senescence, and proliferation effect vary considerably (Spivakov and Fisher, 2007). Furthermore, Bmi-1 expression has been identified as a prognostic factor only in certain types of cancer. For instance, in gliomas, Bmi-1 expression is prognostic in oligodendroglial tumours, in which its expression is abundant, whereas in the much more aggressive high-grade astrocytomas and glioblastomas, Bmi-1 expression is frequently very low and does not correlate with prognosis (Häyry *et al*, 2008). The apparent difference between the role of Bmi-1 in reported nasopharyngeal carcinoma cases (Song *et al*, 2006) and our material of OSSC may also be explained by the fact that nasopharyngeal carcinoma is a non-keratinising type of carcinoma. Tongue carcinoma, on the other hand, is a keratinising SCC and its behaviour may be different from other cancer types.

C-myc expression in a microarray material has been lower in oral carcinomas, compared with laryngeal or pharyngeal primary carcinomas (Freier *et al*, 2003). This result may allude to the fact that oral carcinomas are different diseases compared with their counterparts in other anatomical sites. C-myc is presumed to take part in early oral carcinogenesis, but according to our study, nuclear c-myc immunoexpression did not show a significant correlation with prognosis, although a tendency was observed. C-myc mRNA downregulation has been shown to correlate with poor prognosis and progression of the disease (Vora *et al*, 2007). In this study, c-myc mRNA was not studied. In fact, Vora *et al* observed an inverse correlation between cytoplasmic c-myc protein expression and tumour stage. In our study, cytoplasmic c-myc expression did not show any correlation with clinical parameters. The discrepancy between these results may be caused by the small sample size (*n*=19) used in the study by Vora *et al* (2007).

In this material, Ki-67 immunostaining did not provide any relevant prognostic information.

Snail has been shown to be active in OSSC and, concurrently, we found a positive expression in all our samples. Snail expression correlated with invasion depth in our material, suggesting a role in the primary invasiveness of OSCC. Indeed, tumour thickness has been found to correlate with metastasis, local recurrence, and survival (Po Wing Yuen *et al*, 2002). However, this chain of events did not directly reflect on the prognostic value of Snail expression, which was not statistically significant. However, there was a tendency for high Snail expression and poor prognosis. Although Snail expression has previously been connected to lymph node metastases and clinicopathological tumour stage in oesophageal SCC (Usami *et al*, 2008), in our data, the high Snail expression was correlated only to histopathological grading (well, moderate, or poor differentiation). According to many studies, the histological grading of OSCC does not correlate with clinical outcome (Keski-Säntti *et al*, 2007). This is in line with our results showing Snail expression correlating with tumour grade but not with prognosis. In OSCC, loss of the cadherin/catenin complex has been linked to the degree of differentiation but not to metastatic disease. This is in accordance with our findings, because Snail overexpression is linked to lowered cadherin/catenin expression (Mahomed *et al*, 2007; Zidar *et al*, 2008).

In conclusion, loss of Bmi-1 immunoexpression seems to correlate with clinical outcome of oral tongue SCC, in contrast to Snail or c-myc immunoexpression. Snail expression correlates with histological grading and depth of invasion. Our results suggest that loss of Bmi-1 might serve as a clinically relevant marker for poor prognosis in OSCC.

## Figures and Tables

**Table 1:** Expression of Bmi-1, C-myc, Snail, and Ki-67 in squamous cell carcinoma of the tongue

	**Number of cases**					
**Immunoexpression scoring**	**None**	**Very low**	**Low**	**Moderate**	**High**	**Total**
Bmi-1	13	25	17	10	5	70
C-myc(n)	27	35	6	2	0	70
C-myc(cp)	20	25	10	11	4	70
Snail	0	0	4	18	47	69^a^
Ki-67	0	13	24	14	16	67^a^

Abbreviations: C-myc(n)=nuclear staining; C-myc(cp)=cytoplasmic staining.

a For these markers, <70 representative samples were available.

The frequency of immunopositive tumour cells is shown. Bmi-1 and Snail expression is nuclear, C-myc expression is presented separately for nuclear and cytoplasmic immunoreactivity. No immunoreactive cells is scored as none, those with <30% positive cells as very low, those with 30–50% as low, 51–80% moderate, and those with over 80% immunoreactive tumour cells are scored as high.
